# Fate of aflatoxins M_1_ and B_1_ within the period of production and storage of Tarkhineh: A traditional Persian fermented food

**DOI:** 10.1002/fsn3.2728

**Published:** 2022-01-10

**Authors:** Leila Moradi, Giti Paimard, Ehsan Sadeghi, Milad Rouhi, Reza Mohammadi, Razieh Noroozi, Saeede Safajoo

**Affiliations:** ^1^ Student Research Committee Department of Food Science and Technology School of Nutrition Sciences and Food Technology Kermanshah University of Medical Sciences Kermanshah Iran; ^2^ Department of Food Science and Technology School of Nutrition Sciences and Food Technology Research Center for Environmental Determinants of Health (RCEDH) Health Institute Kermanshah University of Medical Sciences Kermanshah Iran

**Keywords:** aflatoxin B_1_, aflatoxin M_1_, Persian fermented food, Tarkhineh

## Abstract

The objective of the study was to assess the amount of aflatoxin M_1_ (AFM_1_) and aflatoxin B_1_ (AFB_1_) during fermentation, drying, and storage of Tarkhineh—a traditional Persian fermented food—over four months. Tarkhineh samples were produced based on a traditional method. Various concentrations of AFB_1_ (2.5, 5, 7.5, and 10 µg/kg) and AFM_1_, stood at 0.25, 0.5, 0.75, and 1 µg/kg, were added to Iranian yogurt drink, called doogh, samples. Tarkhineh samples were evaluated for AFB_1_ and AFM_1_ on days 0, 2, 6, and 8 and also after drying and four months of storage. In cases of repeatability, recovery, and reproducibility, the high‐performance liquid chromatography through fluorescence detector (HPLC‐FD) method was successfully done to demonstrate aflatoxins (AFs) in Tarkhineh samples. The fermentation process had a considerable consequence on the reduction in AFM_1_ and AFB_1_ as compared to the control group, evidenced by 65.10%–81.20% and 55.80%–74.10%, respectively, after eight days of fermentation (*p* < .05). The highest reduction in AFB_1_ existed in samples containing 2.5 µg/kg toxin, followed by 5, 7.5, and 10 µg/kg, respectively. A similar trend was found for AFM_1_, as the highest concentration was found in samples containing 0.25 µg/kg toxin, followed by 0.5, 0.75, and 1 µg/kg, respectively.

## Graphical abstract

The objective of the study was to assess the amount of aflatoxin M1 (AFM1) and aflatoxin B1 (AFB1) during fermentation, drying, and storage of Tarkhineh—a traditional Persian fermented food—over four months. Our findings indicated that the most probable reason for toxin reduction was the presence of starter culture microorganisms and also low pH of doogh during fermentation.

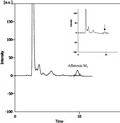

## 1 INTRODUCTION

In 21st century, food products containing aflatoxins above the maximum residue limit have been the center of debate throughout the world since they endanger human healthy lifestyle and commodities international trade (Amirahmadi et al., 2018; Amirkhizi et al., 2015; Moradi et al., 2021). Thus, the international scientific communities, such as the World Health Organization (WHO), Food and Drug Administration (FDA), the Codex Alimentarius Commission the U.S., and the European Commission (EC), have strived substantially to promote analytical procedures besides more stringent regulatory limitations and legislations to guarantee the food safety (Douny et al., 2013; Rastegar et al., 2017).

Aflatoxins, regarded as secondary metabolites of *Aspergillus flavus*, *A. nomius,* and *A. parasiticus,* are discovered in a wide range of animal feeds (barley, straw, alfalfa hay, and corn) and agricultural goods (cereals, nuts, spices, and dried fruits) during growth, harvest, and particularly storage (Fakhri et al., 2019; Noroozi et al., 2020; Prandini et al., 2009; Rahimi et al., 2018). Among eighteen aflatoxin compounds, aflatoxin B_1_ (AFB_1_) is the highest frequent mycotoxin that existed in foods for both humans and animals (Babakhanian et al., 2015; Iqbal et al., 2013). Due to the highly cytotoxic, mutagenic, immunosuppressive, teratogenic, and carcinogenic effects of AFB_1_, being recognized as the first group of human carcinogen, defined in International Agency for Research on Cancer (IARC). To protect consumers’ health, the maximum authorized amount of AFB_1_ in foodstuffs, as well as cereals and breakfast cereals, was considered to be 5 and 2 µg/kg among the European Commission, respectively (European Commission, 2006).

The cytochrome P450 enzyme system metabolizes AFB_1_ into aflatoxin M_1_ (AFM_1_) in the lactating animals’ liver, feeding on AFB_1_‐contaminated diet (Fallah et al., 2011). According to IARC, AFM_1_ has been regarded as the second group of human carcinogens and is observed as a vexingly complicated issue, considering food hygiene (IARC, 2002). AFM_1_ is frequently found in pasteurized, raw, and ultrahigh‐temperature (UHT) milk and dairy outputs, including butter, yogurt, ice cream, cream, and cheese in many countries around the world (Fallah, 2010; Fallah et al., 2009; Hassan & Kassaify, 2014; Iqbal et al., 2017; Škrbić et al., 2015; Zheng et al., 2013; Zinedine et al., 2007). Because of the health‐related problems of AFM_1_, over sixty countries have set up the highest authorized amount for AFM_1_ distributed among dairy productions based on their economic conditions and development (Kos et al., 2014; Nabizadeh et al., 2018). The countries of the European Commission, Turkey, Argentina, Honduras, and also Iran have set up the level permitted with 50 ng/kg in infant, raw, and pasteurized milk (Codex Alimentarius Commission, 2001). In the past decades, several approaches such as fermentation, heating, and irradiation and also the addition of chlorinating, oxidizing, hydrolytic compounds, and medicinal plants have been used to decontaminate food products from AFM_1_ (Sarlak et al., 2017).

Tarkhineh, Tarkhowana or Doowina in Kurdish language, is one of the locally made foods in the western part of Iran (Kermanshah, Ilam, Kurdistan, and Lorestan), produced throughout heating process and fermenting wheat meal, medicinal plants (mint, pennyroyal, and ziziphora), and doogh fermented for approximately a week and then proceeded by sun‐drying for 3–4 days (Bahrami et al., 2016). The doogh is a traditional Persian dairy drink that prepared from churning yogurt in a vessel comprising the potable water, salt, and spices, and definitely, it is one of the most popular beverages in Iran and some other Middle East countries (Bahrami et al., 2016; Sarlak et al., 2017). In a lately represented study done by Bahrami et al. (2016), the medium level concentration of AFM_1_ in samples of Tarkhineh from Kermanshah, Kurdistan, Ilam, and Lorestan provinces was recorded to be 11.0 ± 1.2 ng/kg. The contamination of doogh, wheat, and spices such as mint, pennyroyal, and ziziphora is the potential reason for the existence of AFM_1_ and AFB_1_ in Tarkhineh. In another study, Sarlak et al. (2017) indicated that incorporating 9 log CFU/ml probiotic *Lactobacillus acidophilus* into the fermented doogh significantly reduced its AFM content (0.5 µg/kg) compared with the control group. A similar AFM_1_ reduction in fermented milk during production and storage was reported (Arab et al., 2012). Based on this study, no permissible limit has been established for AFM_1_ in Tarkhineh in Iran, given that Tarkhineh is commonly consumed in winter and autumn in different cities of Iran and is produced by households in rural areas under unsuitable hygienic conditions. According to our knowledge, there is no information on the fate of AFM_1_ and AFB_1_ within the period of production and storage of Tarkhineh. Therefore, it is highly important to analyze the fate of AFM_1_ and AFB_1_ within producing and storing Tarkhineh. Thus, the study presented was conducted with the aim of determination of the occurrence of AFM_1_ and AFB_1_ during fermentation, drying, and storage of Tarkhineh over four months.

## 2 MATERIALS AND METHODS

### 2.1 Materials

AFB_1_ and AFM_1_ standards were bought from Sigma‐Aldrich (UK) with purity certified greater than 99%. AFLA‐Test immunoaffinity column for extract clean‐up was purchased from LC Tech GmbH (Dorfen, Germany) and utilized according to the guidelines of the manufacturer. All chemicals used in this investigation were of HPLC grade and obtained from Merck (Darmstadt, Germany). The deionized water was applied in all procedures. The wheat and raw milk samples were grasped from the regional markets in Kermanshah, located in the western part of Iran. *Lactobacillus delbrueckii* subsp. *bulgaricus* and *Streptococcus salivarius* subsp. *thermophilus* were ordered from the microbial collection of Iranian Research Corporation for Science and Technology (Tehran, Iran). De Man, Rogosa, and Sharpe (MRS) agar was obtained from Merck (Germany).

### 2.2 Preparation of aflatoxins M_1_ and B_1_

The stock solutions of AFB_1_ and AFM_1_ were prepared via dissolving 0.5 mg in 10 ml chloroform and 5 µg in 5 ml acetonitrile, respectively, and consequently, they were stored in −20°C in an amber flask, preventing photodegradation. The appropriate concentrations AFB_1_ and AFM_1_, considered as the aqueous working solutions, were obtained from the stock solutions to an appropriate volume of methanol: water 40:10 v/v, ranging from 0.02 to 20 µg/kg for AFB_1_ and from 0.125 to 2 µg/kg for AFM_1_, which were then kept at refrigerator temperature (4 ± 1°C).

### 2.3 Analysis of HPLC

High‐performance liquid chromatography (HPLC) utilized in this paper is performed via a KNAVER HPLC accompanied with a fluorescence detector (FD detector, model RF‐20A) and LCTech postcolumn photochemical derivatization of UV system. The HPLC system consisted of a C18 analytical column with 250 4.6 mm I.D., 5 mm.

All HPLC analyses were done under isocratic conditions by the use of a mobile phase composed of a combination of ultrapure water: acetonitrile (90:10 v/v, AFB_1_) with a flow rate of 1.5 ml/min and acetonitrile: methanol: water (20:20: 60 v/v, AFM_1_) with a flow rate of 1.2 ml/min. The wavelengths of the FD were set at 329 and 460 nm for the excitation and emission of AFB_1_ and at 365 and 455 nm for the excitation and emission of AFM_1_, respectively. The column temperature was held at 40°C for AFB_1_ and 30°C for AFM_1_. The standard and sample injection solution volumes were 20 μl.

### 2.4 Preparation of Tarkhineh samples

Tarkhineh samples were manufactured based on a traditional method as published in a previous study (Mashak et al., 2014). An amount of 1000 g wheat meal was soaked in 4000 ml sour doogh (a beverage produced by beating unflavored yogurt until it is smooth) and then fermented for 8 days. Subsequently, 20 g dried *Mentha longifolia* powder and 20 g salt were incorporated into the dough‐like mixture. Eventually, it was exposed to sunlight in tiny parts to dry.

### 2.5 Proximate composition of Tarkhineh samples

Moisture, protein, fat, and ash contents of Tarkhineh samples were measured by a standard method (AOAC, 1995). Compositional values are reported on a percent (%) basis.

### 2.6 Spiking of Tarkhineh samples

The initial counts of AFB_1_ and AFM_1_ in the raw milk and wheat were clarified by using HPLC‐FD, as described in Section 3. AFB_1_ and AFM_1_ were not detected in the raw milk and wheat samples. Various concentrations of AFB_1_, which include 2.5, 5, 7.5, and 10 µg/kg, and AFM_1_, which include 0.25, 0.5, 0.75, and 1 µg/kg, were added to the doogh samples. Tarkhineh samples were evaluated for the existence of AFB_1_ and AFM_1_ on days 0, 2, 6, and 8 and also after drying and four months of storage.

### 2.7 Solid‐phase extraction

In order to perform the clean‐up procedure, 25 g of each sample was combined with 0.5 g NaCl. After adding 50 ml ultrapure water: methanol (40:10 v/v), 14 ml of the resultant solution was combined with 86 ml phosphate‐buffered saline. Then, extracts of the sample were carefully moved through C18 SPE column. This trend was proceeded with ultrapure water: methanol (40:10 v/v). Following this, being washed with 10 ml water, the column was dried by N_2_ gas. The resulting residue was dissolved in 2 ml methanol, transmitted to an HPLC vial, and analyzed by HPLC‐FD (Sarlak et al., 2017).

### 2.8 Validation of HPLC method

Considering the validation procedure for aflatoxin residues distributed among animal‐related products, described by the European Communities (EC), the method validation was implemented (European Communities, 2002). Linearity, repeatability, limit of detection (LOD), and limit of quantification (LOQ) were examined in this study. Accordingly, a signal‐to‐noise ratio (S/N) of 3 and 10, LOD, and LOQ were ascertained, where the samples were spiked with different concentrations of AFs (Bahrami et al., 2016). The linearity was determined by injecting different concentrations of AFM_1_ and AFB_1_, respectively, at 0.125–2 and 0.02–20 µg/kg. The percent of recovery was also calculated to confirm the accuracy of the method. Within‐day (run‐to‐run) precision of the HPLC‐FD method, considered as RSD%, was calculated by extracting and analyzing AFs in one sample three times in the same condition.

### 2.9 Survival of Lactobacillus bulgaricus and Streptococcus thermophilus in Tarkhineh samples

Enumerations of *L. bulgaricus* and *S. thermophilus* in the stored Tarkhineh samples were conducted using MRS agar. Then, the plates were incubated within the period of 48 hr, at 37 ± 1°C for under microaerophilic conditions (Jay et al., 2005).

### 2.10 pH determination of Tarkhineh samples

For pH measurement, 5 g of the samples was homogenized with double‐distilled water. 5 min later, pH was examined via a pH meter (Metrohm digital pH meter model 632; Switzerland) at room temperature.

### 2.11 Statistical analysis

SPSS 21 for Windows (SPSS, Chicago, IL, USA) software package was applied to the analysis. The samples were analyzed in triplicate. The significant differences among the means were firstly examined by two‐way analysis of variance (ANOVA) and then via Duncan's multiple range tests to evaluate the treatments (*p* < .05). For evaluation of each toxin, the two‐way repeated‐measures analysis of variance including a within‐subject factor (six levels of storage time) and a between‐subject factor (four different treatments) was applied.

## 3 RESULTS AND DISCUSSION

### 3.1 Exact composition of Tarkhineh samples

Fat, moisture, protein, and ash contents of Tarkhineh samples were measured to be 1.23%, 3.12%, 14.57%, and 7.45%, respectively. The obtained outputs are in agreement with what was done in the previous studies (Mashak et al., 2014; Tabatabaei‐Yazdi et al., 2013).

### 3.2 Validation study

**FIGURE 1:**
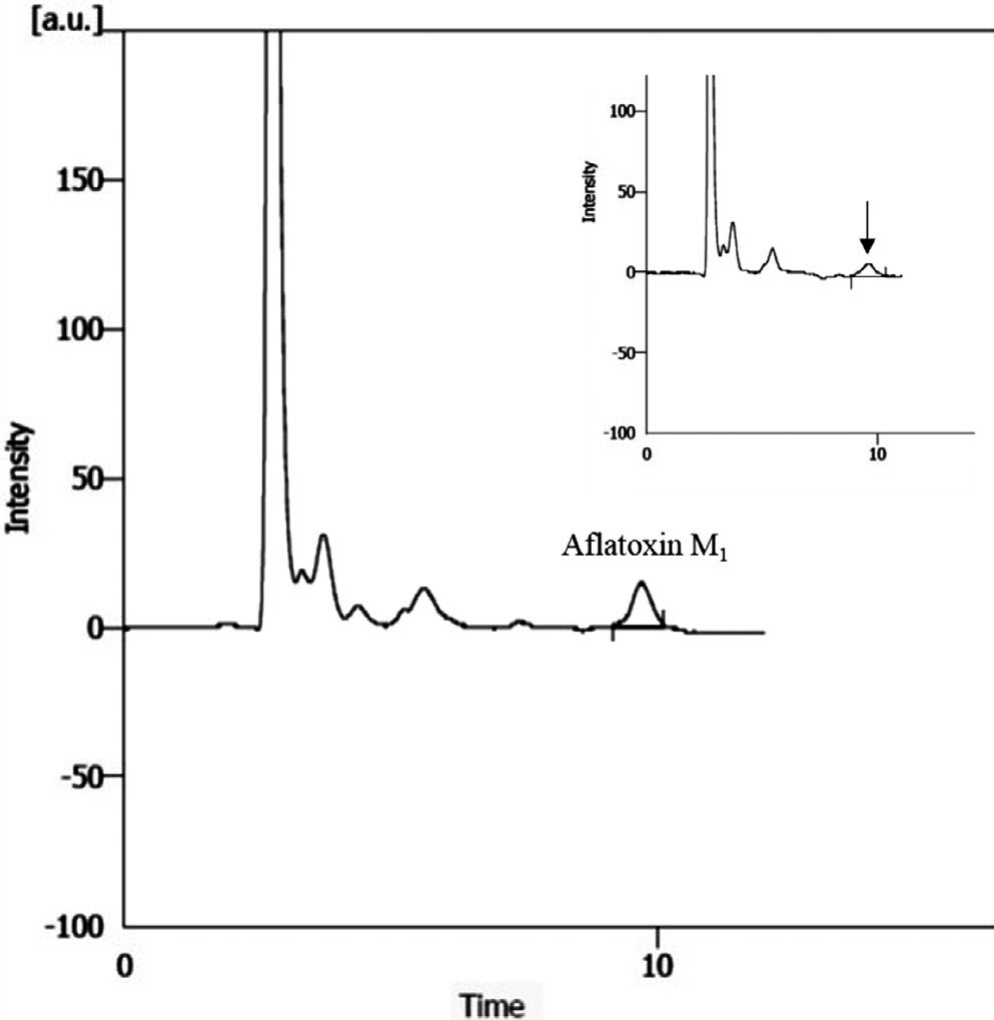
HPLC chromatograms of AFM_1_ standard (2 µg/kg). Inset is the chromatogram of the sample

**FIGURE 2:**
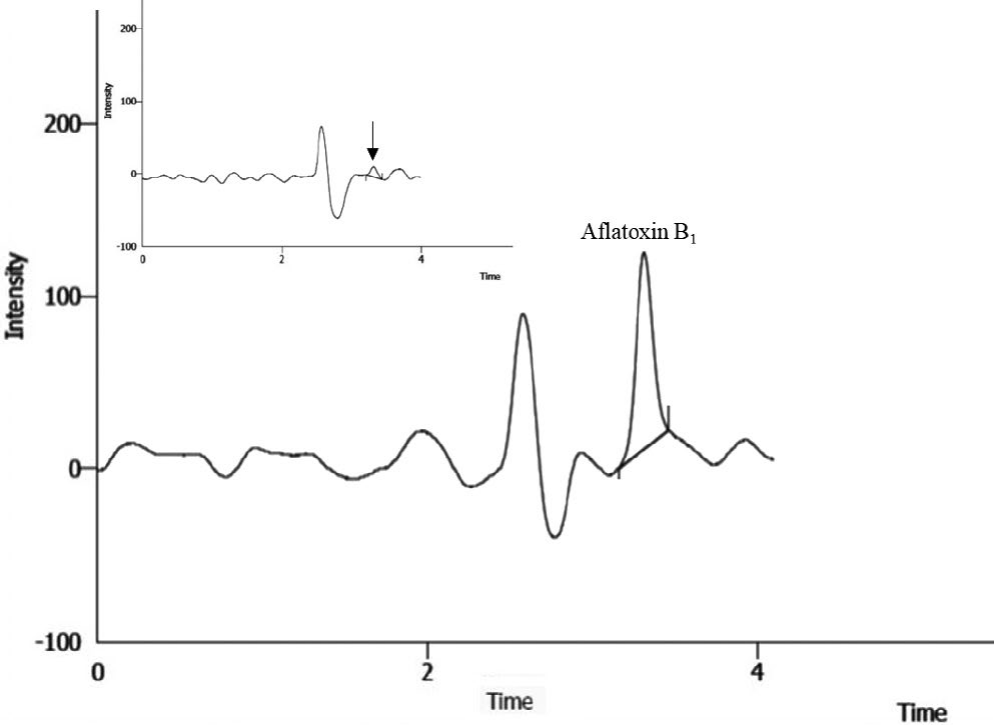
HPLC chromatograms of AFB1 standard (20 µg/kg). Inset is the chromatogram of the sample

The HPLC chromatograms of AFM_1_ and AFB_1_ standards, and their samples as well, are depicted in Figures 1 and 2, respectively. According to the performance of the HPLC‐FD method, the current method in this study was evaluated based on the recovery percentage, LOD, LOQ, *r* ^2^, RSD, and linearity. With regard to our results, the curve of calibration for AFM_1_ and AFB_1_ was linear at disparate concentrations between 0.02 and 20 µg/kg for AFB_1_ and ranging from 0.125 to 2 µg/kg for AFM_1_. The linear regression equations of AFB_1_ and AFM_1_ were *y* = 39.359 *x* + 14.592 and *y* = 47.805 *x* + 4.6708, respectively. These revealed a linear connection between the peak area and the corresponding concentrations of AFB_1_ and AFM_1_. There was a significant correlation between results and the concentration of AFB_1_ injected, in which the calculated coefficient of determination (*r* ^2^) was 0.996. Moreover, LOD, 0.005 µg/kg, and LOQ, 0.015 µg/kg, were found. The *r* ^2^, LOD, and LOQ for the injected AFM_1_ were 0.9937, 0.02, and 0.045 µg/kg, respectively. The comparison of the obtained results with the performance standard introduced in the Commission Regulation (EC) No 401/2006 exhibited the method that met the performance criteria in terms of repeatability, recovery, and reproducibility for AFs in Tarkhineh samples.

### 3.3 Fate of aflatoxins M_1_ and B_1_ during Tarkhineh fermentation and storage

The results of the fate of AFB_1_ and AFM_1_ during Tarkhineh fermentation are presented in Tables 1 and 2, respectively. Based on our findings, the fermentation process had an immense consequence on the reduction in AFM_1_ and AFB_1_ compared with the control group, evidenced by 65.10%–81.20% and 55.80%–74.10%, respectively, after eight days of fermentation (*p* < .05). Moreover, the reduction percentage of toxins significantly varied with toxin concentrations in Tarkhineh samples (*p* < .05). In detail, the highest reduction in AFB_1_ existed in samples containing 2.5 µg/kg toxin, followed by 5, 7.5, and 10 µg/kg, respectively. A similar trend was also found for AFM_1_, as the highest concentration was examined in samples containing 0.25 µg/kg, which was followed by 0.5, 0.75, and 1 µg/kg, respectively. This is probably because of the practice of *S*. *thermophilus* and *L. bulgaricus*, which can be mainly related to weak noncovalent bound interactions, like pertaining to hydrophobic pockets on the bacterial surface (Campagnollo et al., 2016; Iqbal et al., 2015).

**TABLE 1:** Fate of aflatoxin B_1_ during Tarkhineh production and storage

Spiked level	Time					
	Day 0	Day 2	Day 6	Day 8	After drying	Month 4
2.5 μg/kg	1.45 ± 0.02^Aa^	1.33 ± 0.06^Aa^	1.04 ± 004^Aa^	0.65 ± 0.01^Ab^	0.95 ± 0.01^Aa^	0.94 ± 0.01^Aa^
5 μg/kg	3.17 ± 0.01^Ba^	2.87 ± 0.03^Ba^	2.34 ± 0.01^Ba^	1.58 ± 0.02^Bc^	1.98 ± 0.03^Bb^	1.99 ± 0.03^Bb^
7.5 μg/kg	5.40 ± 0.04^Ca^	4.45 ± 0.02^Ca^	3.55 ± 0.04^Cb^	2.88 ± 0.01^Cc^	3.15 ± 0.02^Cb^	3.12 ± 0.02^Cb^
10 μg/kg	8.10 ± 0.04^Da^	6.65 ± 0.05^Db^	5.56 ± 0.07^Dc^	4.42 ± 0.01^Dd^	5.25 ± 0.01^Dc^	5.23 ± 0.01^Dc^

Note

For each sampling day, different capital letters among groups indicate significant differences (*p* < .05). Means with different lowercase letters in the same row are significantly different (*p* < .05).

**TABLE 2:** Fate of aflatoxin M_1_ during Tarkhineh production and storage

Spiked level	Time					
	Day 0	Day 2	Day 6	Day 8	After drying	Month 4
0.25 μg/kg	0.16 ± 0.01^Aa^	0.12 ± 0.03^Aa^	0.09 ± 0.01^Ab^	0.04 ± 0.01^Ab^	0.12 ± 0.02^Aa^	0.12 ± 0.04^Aa^
0.5 μg/kg	0.34 ± 0.01^Aa^	0.25 ± 0.02^Aa^	0.19 ± 0.03^Ba^	0.12 ± 0.02^Ba^	0.24 ± 0.03^Ba^	0.23 ± 0.03^Aa^
0.75 μg/kg	0.57 ± 0.05^Ba^	0.42 ± 0.02^Ba^	0.34 ± 0.01^Ca^	0.22 ± 0.01^Cb^	0.36 ± 0.03^Cc^	0.36 ± 0.01^Bc^
1 μg/kg	0.83 ± 0.03^Ca^	0.65 ± 0.03^Ca^	0.51 ± 0.03^Da^	0.35 ± 0.01^Db^	0.46 ± 0.01^Cc^	0.46 ± 0.01^Cc^

Note

For each sampling day, different capital letters among groups indicate significant differences (*p* <.05). Means with different lowercase letters in the same row are significantly different (*p* <.05).

El‐Khoury et al. (2011) evaluated the capability of some strains of lactic acid bacteria, especially *S. thermophilus* and *L. bulgaricus,* to remove AFM_1_ within the period of yogurt production. Their findings indicated that *S. thermophiles* and *L. bulgaricus* bound 70% and 87.6% of AFM_1_, respectively. Further, reported 77%–99% AFM_1_ was eliminated by *Lactobacillus* spp. in an extensive and medium amount (Turbic et al., 2002). Our findings are also in agreement with those reported for Feta cheese (Motawee & McMahon, 2009), acidophilus milk (Khaneghah et al., 2017), yogurt (Govaris et al., 2002; Montaseri et al., 2014; Sevim et al., 2019), doogh (Sarlak et al., 2017), and buttermilk and kefir (Wiseman & Marth, 1983). The reduction in AFB_1_ in nondairy products such as pistachio nuts (Rastegar et al., 2017), peanut and olive oils (Fan et al., 2013), and corn kernels (Hojnik et al., 2021) was also reported.

The viability of *S. thermophilus* and *L. bulgaricus* during the production of Tarkhineh sample was also evaluated. According to our findings, the total viable counts of *L. bulgaricus* and *S. thermophilus* were 7.88 ± 0.04–9 ± 0.02 log CFU/ml, 7.45 ± 0.05 log CFU/g, and 6.45 ± 0.07 log CFU/g during fermentation, drying, and storage of Tarkhineh samples, respectively (Table 3). The reduction in *S. thermophilus* and *L. bulgaricus* can be attributed to the acidic condition of the samples during fermentation (Tsakalidou & Papadimitriou, 2011). The survival of bacterial cells during the drying and storage of samples can be originated from the adhesion forces between food matrices and the strains and finally overcome the limitations, including the physicochemical and osmotic conditions (Jay et al., 2005). Our outcomes are in agreement with those reported for Feta cheese (Mohammadi et al., 2020), doogh (Sarlak et al., 2017), milk (Abdelmotilib et al., 2018; Serrano‐Niño et al., 2013), and peanut grains (Silva et al., 2015).

**TABLE 3:** Fate of *L. bulgaricus* and *S. thermophilus* during Tarkhineh production and storage

	Time					
	Day 0	Day 2	Day 6	Day 8	After drying	Month 4
Count (log CFU/ml)	9.00 ± 0.02^A^	8.12 ± 0.04^B^	8.09 ± 0.05^B^	7.88 ± 0.04^B^	7.45 ± 0.05^C^	6.45 ± 0.07^C^

Note

Means with different capital letters in the same row are significantly different (*p* < .05).

Furthermore, contributors, such as low pH and the formation of organic acids and other fermentation by‐products, result in the reduction in AFM_1_ (Bahrami et al., 2016). The consequences of this study exhibited that the pH values of Tarkhineh samples were declined within fermentation and drying and storage steps (Table 4). In the current study, a definite link was observed between pH reduction and AFM_1_ and AFB_1_ decomposition. The previous studies also reported that AFM_1_ and AFB_1_ reduced with a decrease in pH (Hassanin, 1994; Sarimehmetoğlu & Küplülü, 2004), being complied with our findings. In stark contrast, though, Blanco et al. (1993) and Wiseman and Marth (1983) demonstrated that aflatoxins B_1_, B_2_, G_1_, G_2_, M_1_, and M_2_ did not alter during yogurt, buttermilk, and kefir fermentation. Van Egmond et al. (1977) and Munksgaard et al. (1987) found that the concentration of AFM_1_ was increased after the fermentation process, which is in contrast with our findings (Munksgaard et al., 1987; Van Egmond et al., 1977). As indicated in the previous studies, the production of organic acids and other fermentation by‐products can be considered a noteworthy method for detoxification of AFM_1_ and AFB_1_ in food products compared with other chemical approaches.

**TABLE 4:** The pH value of Tarkhineh during production and storage

	Time					
	Day 0	Day 2	Day 6	Day 8	After drying	Month 4
Value	4.45 ± 0.12	4.42 ± 0.03	4.59 ± 0.01	4.81 ± 0.01	4.91 ± 0.11	4.93 ± 0.01

As presented in Tables 1 and 2, the concentrations of AFB_1_ and AFM_1_ significantly increased, recorded by 0.95–5.25 μg/kg and 0.12–0.46 μg/kg during the drying step of the samples, respectively, because *S. thermophilus* and *L. bulgaricus* were reduced by 2.55 log CFU/g and Tarkhineh samples lost their moisture during the drying step, which is in agreement with the results of Pietri et al. (2016) on the fate of AFM_1_ during Parmesan cheese production (Pietri et al., 2016). Manetta et al. (2009) also reported that the AFM_1_ concentration in Grana Padano cheese, ripened for 12 months, was approximately 4‐ to 4.5‐fold more than in the milk (Manetta et al., 2009). Moreover, the results of the presented study demonstrated that the concentrations of AFB_1_ and AFM_1_ were noticeably constant after four months of sample storage at room temperature.

## 4 CONCLUSION

With regard to this study's results, the fermentation of doogh during Tarkhineh production can significantly reduce the concentrations of AFM_1_ and AFB_1_ (*p* < .05). Our findings indicated that the most probable reason for toxin reduction was the low pH of doogh during fermentation and also the presence of starter culture microorganisms, including *L. bulgaricus* and *S. thermophilus* as well as native probiotics in the Tarkhineh to bind the mycotoxins. Accordingly, the fermentation process could remarkably reduce AFB_1_ and AFM_1_ concentrations during fermentation while the concentrations of AFB1 and AFM1 could be constant after four months of sample storage. The obtained results were in an agreement with those reported for milk and dairy outputs.

## CONFLICT OF INTERESTS

There is no conflict of interest found in this study.

## Data Availability

The datasets generated during and/or analyzed during the current study are available from the corresponding author on reasonable request.
